# Concentration-Dependent Protection by Ethanol Extract of Propolis against *γ*-Ray-Induced Chromosome Damage in Human Blood Lymphocytes

**DOI:** 10.1155/2011/174853

**Published:** 2010-10-11

**Authors:** A. Montoro, J. F. Barquinero, M. Almonacid, A. Montoro, N. Sebastià, G. Verdú, V. Sahuquillo, J. Serrano, M. Saiz, J. I. Villaescusa, J. M. Soriano

**Affiliations:** ^1^Servicio de Protección Radiológica, Hospital Universitario La Fe, 46009 Valencia, Spain; ^2^Unitat d'Antropologia Biològica, Departamento de Biologia Animal, Biologia Vegetal i Ecologia, Facultat de Biociències, Universitat Autònoma de Barcelona, 08193 Bellaterra, Spain; ^3^Área de Nutrición y Bromatología, Facultat de Farmàcia, Universitat de València, 46100 Burjassot, Spain; ^4^Departamento de Ingeniería Química y Nuclear, Escuela Superior de Ingenieros Industriales, Universidad Politécnica de Valencia, 46022 Valencia, Spain; ^5^Dietéticos Intersa, Plaza Dr. Seres no. 13, Torreserona, Lleida 25131, Spain

## Abstract

Radioprotection with natural products may be relevant to the mitigation of ionizing radiation-induced damage in mammalian systems; in this sense, propolis extracts have shown effects such as antioxidant, antitumoral, anti-inflammatory, and immunostimulant. We report for the first time a cytogenetic study to evaluate the radioprotective effect, *in vitro*, of propolis against radiation-induced chromosomal damage. Lymphocytes were cultured with increasing concentrations of ethanol extract of propolis (EEP), including 20, 40, 120, 250, 500, 750, 1000, and 2000 *μ*g mL^−1^ and then exposed to 2 Gy *γ*-rays. A significant and concentration-dependent decrease is observed in the frequency of chromosome aberrations in samples treated with EEP. The protection against the formation of dicentrics was concentration-dependent, with a maximum protection at 120 *μ*g mL^−1^ of EEP. The observed frequency of dicentrics is described as negative exponential function, indicating that the maximum protectible fraction of dicentrics is approximately 44%. Free radical scavenging and antioxidant activities are the mechanisms that these substances use to protect cells from ionizing radiation.

## 1. Introduction

Attention has been shifted towards the evaluation of plant products as radioprotectors, in the last 15 years, due to their efficacy and low toxicity. The proposed radioprotective efficacy of plant extracts is a result of their containing a large number of active constituents, such as antioxidants, immunostimulants, and compounds with antimicrobial activity. Therefore, screening herbal drugs offers a major focus for new drug discovery [1]. Propolis is a strongly adhesive and resinous substance transformed and used by bees to seal holes in their honeycombs, smooth out the internal walls, and protect the entrance against intruders. It is a product of great interest, both in the field of medicine and the pharmaceutical industry with numerous properties including anti-inflammatory, immunostimulant, hepatoprotector, and carcinostatic [2, 3]. To obtain propolis compounds, the usual manner is to extract the soluble fraction with alcohol, being the most common ethanol extract of propolis (EEP). As compared with a water-soluble derivative of raw propolis (WSDP), EEP contains a higher proportion of lypophilic compounds from the flavonoid-aglycones class, such as flavones and flavonols, and flavanones [4]. More than 200 constituents have been identified so far from propolis [5].

Damaging effects of ionizing radiation on DNA are brought about by both direct and indirect mechanisms. Direct action produces disruption of chemical bonds in the structure of DNA while indirect effects result from highly reactive free radicals such as ^•^OH, ^•^H, and *e* ^−^_aq_ mainly produced during the radiolysis of water, and their subsequent interaction with DNA. It is also known that compounds able to interact with the induced free radicals, so-called “scavengers”, have protective effects against radiation-induced DNA damage. The identification and development of effective, nontoxic, radical scavengers, which can protect against genetic damage by radiation in humans, is of great interest [6]. Unfortunately, most of chemical radioprotectors (AET, WR 2721, WR 1065) have shown toxic side effects that limit their use in medical practice [4]. Propolis has shown protect against the induction of apoptosis by radiation; since it is an imunomodulator, it has antioxidative and cytotoxic activities, antitumor activities, and scavenges free radicals produced by the indirect effect of ionizing radiation [2, 7–12]. Moreover, several reports have demonstrated the ability of EEP to protect mice against gamma irradiation preventing exaggerated inflammatory response [9], promoting high survival in mice [10], protecting their whole blood cells, and also diminishing primary DNA damage in mice [4, 13].

Chromosome aberrations have been used as a sensitive monitor of DNA damage in studies of several radioprotectors [6, 14, 15]. Recently, we have reported that human peripheral blood lymphocytes *in vitro* pretreated with 1000 *μ*g·mL^−1^ of EEP and then exposed to gamma radiation, exhibited a significantly reduced incidence of chromosome aberrations [16].

The aim of this study is to assess the radioprotective effect of different concentrations of propolis to modulate the frequency of radiation-induced chromosome aberrations. To date, it is first article, as *in vitro* study, to evaluate the concentration-dependent protection of propolis against induction of chromosome aberrations by ionizing radiation in human.

## 2. Materials and Methods

### 2.1. Propolis Extraction Procedure and Concentration of Phenolics in the Extract

Ethanolic extract of propolis (EEP) was prepared and analyzed according to Kosalec et al. [17] and Sobočanec et al. [18]. Briefly, raw propolis (10 g), supplied by Dietéticos Intersa S.A (Lleida, Spain), was crushed into small pieces in a mortar and mixed vigorously with 50 mL of 80% ethanol at 37°C for 48 hours being the stock of EEP of 200000 *μ*g mL^−1^ filtered through Whatman no. 4 paper, lyophilized, and kept in dark at 4°C [17]. 

Chromatographic analysis from EEP was performed on an HPLC system (LaChrom L-7100 Series) equipped with a quaternary pump, multiwave UV/vis detector, autosampler and fraction collector. The analytical column was a Spherisorb ODS-2 (250 × 4.6 mm I.D., 5 *μ*m) (Altex. Scientific, Inc., Berkeley, CA, USA) [18]. The stock of EEP has the following flavonoids and phenolic acids (chrysin, 14100 *μ*g mL^−1^; apigenin, 14900 *μ*g mL^−1^; acacetin, 15800 *μ*g mL^−1^; galangin, 14900 *μ*g mL^−1^; kaempferol, 15800 *μ*g mL^−1^; kaempferide, 16100 *μ*g mL^−1^; quercetin, 16700 *μ*g mL^−1^; cinnamic acid, 8200 *μ*g mL^−1^; *o*-coumaric acid, 9100 *μ*g mL^−1^; *m*-coumaric acid, 9100 *μ*g mL^−1^; *p*-coumaric acid, 9100 *μ*g mL^−1^; caffeic acid, 9900 *μ*g mL^−1^; CAPE, 15700 *μ*g mL^−1^).

From the stock solution, and using ethanol as solvent, EEP was added to 12 mL of human peripheral blood samples to final concentrations of 20, 40, 120, 250, 500, 750, 1000, and 2000 *μ*g mL^−1^, in all cases the volume added was 250 *μ*L. The concentration of 1000 *μ*g mL^−1^ was considered as positive control [16], and two negative controls were evaluated, one only with 250 *μ*L of ethanol, and another without any treatment.

### 2.2. Irradiation Conditions

Human peripheral blood samples were collected, after their informed consent, in sterile vacutainer tubes (Becton, Dickinson and Company, Franklin Lakes, NJ, USA) containing lithium heparin as anticoagulant. The different concentrations of EEP were added 1 hour before irradiation and incubated for 1 hour at 37°C. Blood samples were irradiated at 2 Gy (dose rate 50 cGy min^−1^) using a Cobalt Teletherapy Unit located at Hospital La Fe (Valencia). In addition, one blood sample in which 1000 *μ*g mL^−1^ of EEP was added just after the irradiation was also done. A tube with peripheral blood sample was irradiated to 2 Gy in the presence of 95% ethanol and without EEP. In order to ensure a homogeneous irradiation, the International Atomic Energy Agency (IAEA) recommendations [19] were followed during the irradiations.

### 2.3. Culture Conditions

For each treatment, separate cultures were set up by mixing 0.75 mL of whole blood with 5 mL of PB-Max Karyotiping medium (Gibco, Barcelona, Spain) and incubated 48 hours at 37°C. To analyze exclusively first-division metaphases, a final concentration of 12 *μ*g mL^−1^of bromodeoxyuridine (Sigma, Madrid, Spain) was present since the setting up of the cultures. According to IAEA (1986), 150 *μ*g of Colcemid (Gibco, Barcelona, Spain) was added 2 hours before harvesting.

### 2.4. Cytogenetic Analysis

Two- to three-day-old slides were stained with Fluorescence plus Giemsa stain technique [20]. Chromosomal analysis was carried out exclusively on first-division metaphases containing 46 centromeres. Chromosomal abnormalities were classified as follows: dicentric chromosomes (dic) and rings (r) only scored when an acentric fragment was present. Acentric fragments, not associated with dicentric and ring chromosomes, were classified as extra acentric fragments (ace). Translocations and inversions were only recorded when the morphology of the derivative chromosome was clearly indicative of this kind of rearrangement. Other abnormalities like chromatid breaks (chtb) and gaps were also recorded. According to IAEA, [19] and taking into account that after gamma irradiation, the cell distribution of dicentrics follows a Poisson, and in order to have the same accuracy in all EEP treatments, the number of analysed cells was those needed to score 100 dicentrics.

### 2.5. Statistical Analysis

For statistical analysis, Student *t*-test was used and *P*-values <.05 were considered significant. Correlation was assessed using Spearman's rank correlation coefficient. The Poisson distribution was checked by the test quantity *U* of the dispersion index (variance/mean) [21]. All statistical analyses were carried out using SPSS (Statistical Package for Social Sciences) version 10.0 for Windows.

## 3. Results

### 3.1. Chromosome Aberrations in Human Lymphocytes Exposed at 2 Gy of *γ*-Ray in Different Conditions

**Figure 1:**
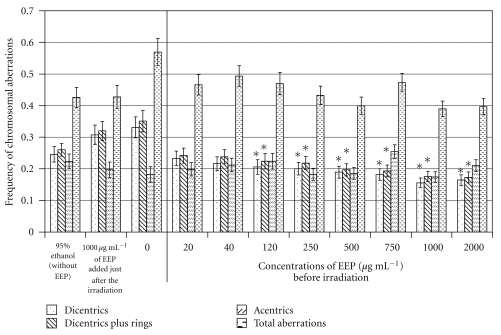
Chromosome aberrations in human lymphocytes exposed to 2 Gy of gamma radiation and different conditions: only in the presence of 250 *μ*L of the ethanol (without EEP), with a concentration of 1000 *μ*g mL^−1^ of EEP added just after the irradiation, without any treatment, concentration 0, and with increasing concentrations of EEP (from 20 to 2000 *μ*g mL^−1^) added before irradiation. (*) Concentrations with values significantly different from the results obtained in the blood irradiated without any treatment.

Figure 1 shows the following cytogenetic results: 95% ethanol (without EEP), 1000 *μ*g mL^−1^ of EEP applied after the irradiation, value of irradiated lymphocytes with 2 Gy with different concentrations, from 0 to 2000 *μ*g mL^−1^, of EEP before irradiation.

After 2 Gy irradiation, the frequency (±SE) of dicentrics without any treatment (0.33 ± 0.03) was higher than that observed when blood was irradiated in presence of ethanol (0.25 ± 0.02). When 1000 *μ*g mL^−1^ of EEP was administrated just after the irradiation (0.31 ± 0.03), no differences with respect to the sample without any treatment were observed. When considering dicentrics plus rings or extra acentric fragments instead of dicentrics, similar results were obtained. Others chromosome aberrations, including chromatid breaks, gaps, translocations, and inversions, were studied but they were not statistically significant.

When the different concentrations of EEP administered 1 hour before irradiations were evaluated, the frequency of dicentrics in all concentrations was lower than that observed in the untreated sample. Moreover, there is a significant negative correlation between the concentration of EEP and the frequency of dicentrics. The decrease with respect to the untreated sample was significant since the 120 *μ*g mL^−1^ concentration of EEP (0.33 ± 0.03 versus 0.21 ± 0.02) to the 2000 *μ*g mL^−1^ (0.16 ± 0.02). Similar results were obtained when dicentrics plus rings were considered. However, for extra acentric fragments, this radioprotective effect was not found.

### 3.2. Poisson Distribution

The distribution of cells containing a different number of dicentrics and the frequencies is shown in Table 1, and in all cases follows a Poisson distribution. The intercellular distribution of dicentrics and dicentrics plus rings follows a Poisson distribution in all cases with or without propolis. Departures from Poisson were assessed in terms of the test quantity *U* [21], a value of *U* > 1.96 indicates overdispersion at the 5% level of significance.

### 3.3. Negative Exponential Function

**Figure 2:**
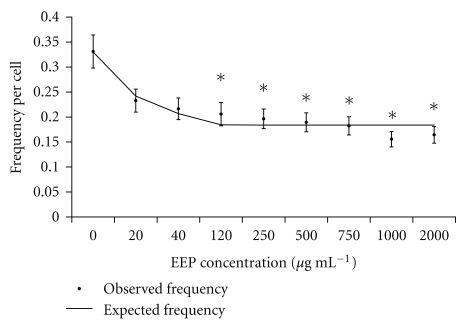
For each concentration of EEP, there observed frequencies of dicentrics (±SE) after 2 Gy irradiation. Solid line represents the expected frequency by the negative exponential function obtained. (*) Concentrations with values significantly different from the results obtained in the blood irradiated without any treatment.

At the highest concentrations of EEP, saturation in the frequency of dicentrics was observed (Figure 2). The observed frequencies of dicentrics were fitted by negative exponential function (see (1)) using the maximum likelihood analysis of regression models [14]:

(1) $$\begin{array}{c} \frac{Y\left(C\right)}{Y\left(0\right)}=\left[{\text{NP}}_{f}+P_{f}e^{-\delta C}\right], \end{array}$$

where *Y*(*C*) is the frequency of dicentrics expected after blood irradiation at 2 Gy in a medium supplemented with a *C* concentration of EEP; *Y*(0) is the frequency of dicentrics without EEP treatment; NP_*f*_ represents the fraction of dicentrics nonprotected and *P*_*f*_ the protective fraction of dicentrics. *P*_*f*_ depends on an exponential “decay” parameter *δ*, and on *C*. The obtained function was *Y*(*C*) = *Y*(0)[(0.56 ± 0.03) + (0.44 ± 0.03)*e* ^(−46.14 ± 16.41)*C*^] (Figure 2). There was a good adjustment between the observed values and the expected ones (*χ* ^2^ = 6.3, *d* *f* = 7, *P* = .5). The function estimates that a 56% of the induced frequency of dicentrics cannot be reduced, whilst the remaining 44% can be reduced depending on the EEP concentration, with the maximum reduction reached at 120 *μ*g mL^−1^.

## 4. Discussion

The present study demonstrates the ability of propolis to reduce significantly the radiation-induced chromosome damage in human cells exposed* in vitro* to *γ*-rays. The protection against the formation of dicentrics was concentration-dependent, with a maximum protection beyond 120 *μ*g mL^−1^. Further increase in the concentration of propolis showed no additional protection. The maximum level of radioprotection when lymphocytes were exposed to 2 Gy of gamma rays was around 44% of the initial damage. Differences in the radioprotection between dicentric and acentric chromosomes could be due to the different origin of chromosome or chromatid aberration formation. Therefore, the protection could be placed in chromosomal aberrations with category-type interchange [22]. 

**Figure 3:**
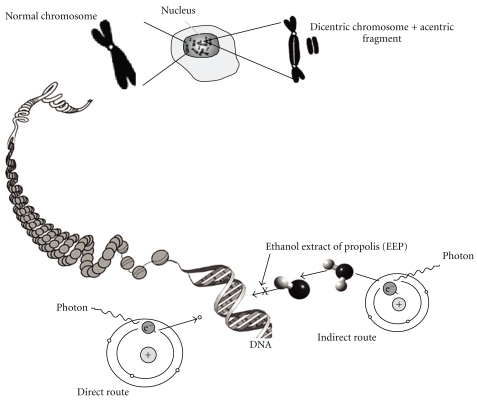
Chromosomal damage, that is, dicentric-acentric fragment production, is done by the direct (radical-induced primary damage) and indirect (reactive free radicals, that is, ^•^OH, produced following radiolysis of water) route action of ionizing radiation. Radiation protection of ethanolic extract of propolis (EEP) is carried out in the indirect route. The X symbol indicates the pathway inhibited by EEP.

In accordance with literature data, the information on the use of EEP for radioprotective capability is limited. The protective effect of propolis against ionizing radiation could be explained by both the direct scavenging of free radicals produced by the indirect effect [9] and the activation of oxidative repair enzymes [23–25]. Both, scavenger and antioxidant properties are involved in the protection against the induction of chromosomal alterations by ionizing radiation (Figure 3).

To avoid the harmful effect of free radicals, radioprotectors must be present during the irradiation. In this sense, if peripheral blood lymphocytes were irradiated at 2 Gy of *γ*-ray and incubated one hour in the presence of EEP (1000 *μ*g mL^−1^), no differences in the frequency of dicentrics were found with respect to untreated samples. These results could indicate that scavenger of free radicals explains better than other mechanisms the radioprotective effect of propolis. In this sense, it has been described that the solvent used in our experiments (ethanol 95%) is known as an efficient scavenger. Sasaki and Matsubara [26] irradiated human blood lymphocytes with 3 Gy of *γ*-rays in the presence of increasing concentrations of ethanol and observed a significant reduction in the frequency of dicentrics. Their results indicated the greatest protection at 1 M. However, although there is some protective effect by the ethanol, the reduction of dicentrics observed in the present study cannot be only attributed to the presence of ethanol. In our experiments, if peripheral blood samples were irradiated in the presence of ethanol (0.3 M) without propolis a slight, but not significant, reduction was observed (Table 1, control: 0.33 ± 0.03 versus 0 + 95% EtOH: 0.25 ± 0.02), but in presence of 2000 *μ*g mL^−1^ the frequency is reduced to 0.16 ± 0.02 dicentrics per cell.

In the present study, the maximum protectable fraction is about a 44%; using the same approach (analyzing the cytogenetic effect), this maximum protectable fraction has been studied with several compounds: with alcohol groups (-OH) (methanol, ethanol, isopropanol, *t*-butanol, etilenglicol, and glicerol), and with sulfhydryl groups (SH) (cystein, cysteamine, and mercaptoethanol). Sasaki and Matsubara [26] found a maximum protectable fraction of 59% for the alcohol groups and of 80% for the sulfhydryl groups. Authors assume that the maximum protectable fraction is related to the indirect action of the ionizing radiation. Other chemical compounds like DMSO at 1 M have a maximum protectable fraction of about a 70% [14]. The active form of amifostine (WR-1065) showed a maximum protective fraction of 87% at 8 mM [15]. The melatonin has also radioprotector effects with a maximum protectable fraction of 68% at 2 mM [6]. Simultaneous treatment with propolis and a chemotherapeutic agent doxorubicin led to a reduction of 64.3% in the frequency of chromosome aberrations [27]. However, most of these products produce serious side effects or cytotoxicity, and some of them are considered to be toxic at the concentrations required to reach radioprotection. Propolis is considered relatively nontoxic and safe at low doses. However, adverse effects are common at doses over 15 g·day^−1^ [2].

Regarding the radioprotective properties of other natural products, Jagetia and Baliga [28] by analyzing micronuclei reported that the use of 12.5 *μ*g mL^−1^ of the leaf extract of *Syzygium cumini* diminishes 3.6 times the effect. When 2000 *μ*g mL^−1^ of *Ginseng* extract is used, a reduction of 46.5% of the induction micronuclei has been described [29]. The observed results for *Ginseng* are similar to those observed in the present study when a reduction of 44% was observed for 120 *μ*g mL^−1^ of EEP. 

The mechanism of action of EEP extract still remains unknown, in part due to the fact that only some out of the 200 constituents of propolis have been identified so far [5]. It has been described that between them, the constituents with major contribution in the radioprotective effect are the flavonoids. Some of the radioprotective mechanisms are scavenger potency against free radicals [30], immunological properties [10], protection against inflammatory responses [9], protection against fetal effects of radiation, and apoptosis in cancer cells [2]. Moreover, Kunimasa et al. [24] have demonstrated that flavonoids have antioxidative potency *in vitro* and *in vivo,* protect mice against lethal effects of whole-body irradiation, and diminish primary DNA damage [4]. We hypothesize that EEP can influence the low frequency of radiation-induced chromosome aberrations due to that inhibit the indirect (radical) mechanism due to that EEP act as radical scavengers (Figure 3).

Further research leading *in vivo* studies are necessary to know if there is an interindividual variability in the protective effect of EEP, an if the concentrations used in *in vitro* studies are toxic for human administration. Studies based on cytotoxicity and cell cycle delay by EEP *in vitro* cultures are ongoing. Also, studies should be focused to determine the individual compounds of propolis responsible for radioprotective effect and their mode of action.

## Figures and Tables

**Table 1:** Dicentric cell distribution and frequencies (*Y* ± *S* *E*), dispersion index (DI) and normalized unit of this index (*U*) for each conditions and with 2 Gy gamma rays.

EEP (*μ*g·mL^−1^)	Cell scored	dic	Cell with 0 dic	Cell with 1 dic	Cell with 2 dic	*Y* ± SE	DI	*U*
0	302	100	216	72	14	0.33 ± 0.03	0.95	−0.59
0 + 95% EtOH^1^	411	101	316	89	6	0.24 ± 0.02	0.88	−1.80
1000^2^	325	100	238	74	13	0.31 ± 0.03	0.94	−0.73

20	438	102	346	82	10	0.23 ± 0.02	0.97	−0.51
40	462	100	370	84	8	0.22 ± 0.02	0.95	−0.83
120	379	78	308	64	7	0.21 ± 0.02	0.98	−0.33
250	509	102	412	92	5	0.20 ± 0.02	0.90	−1.61
500	528	100	436	84	8	0.19 ± 0.02	0.97	−0.45
750	554	101	458	91	5	0.18 ± 0.02	0.92	−1.36
1000	649	101	556	85	8	0.16 ± 0.02	1.00	0.08
2000	590	97	501	81	8	0.16 ± 0.02	1.00	0.04

EEP: Ethanol extract of propolis; dic: dicentrics; *Y* ± SE: Frequencies of dicentrics ± standard errors; DI: Dispersion index (variance/mean); *U*: Normalized Unit of D; ^1^0 + 95% EtOH: without propolis and with 250 *μ*L of ethanol 95%; ^2^1000 after: EEP added just after irradiation.
